# Predicting weighted unobserved nodes in a regulatory network using answer set programming

**DOI:** 10.1186/s12859-023-05429-3

**Published:** 2023-08-25

**Authors:** Sophie Le Bars, Mathieu Bolteau, Jérémie Bourdon, Carito Guziolowski

**Affiliations:** École Centrale Nantes, CNRS, LS2N, UMR 6004, Nantes Université, Nantes, 44000 France

**Keywords:** Regulatory network, Answer set programming, OMIC data integration, Regulatory and metabolic models integration

## Abstract

**Background:**

The impact of a perturbation, over-expression, or repression of a key node on an organism, can be modelled based on a regulatory and/or metabolic network. Integration of these two networks could improve our global understanding of biological mechanisms triggered by a perturbation. This study focuses on improving the modelling of the regulatory network to facilitate a possible integration with the metabolic network. Previously proposed methods that study this problem fail to deal with a real-size regulatory network, computing predictions sensitive to perturbation and quantifying the predicted species behaviour more finely.

**Results:**

To address previously mentioned limitations, we develop a new method based on Answer Set Programming, MajS. It takes a regulatory network and a discrete partial set of observations as input. MajS tests the consistency between the input data, proposes minimal repairs on the network to establish consistency, and finally computes weighted and signed predictions over the network species. We tested MajS by comparing the HIF-1 signalling pathway with two gene-expression datasets. Our results show that MajS can predict 100% of unobserved species. When comparing MajS with two similar (discrete and quantitative) tools, we observed that compared with the discrete tool, MajS proposes a better coverage of the unobserved species, is more sensitive to system perturbations, and proposes predictions closer to real data. Compared to the quantitative tool, MajS provides more refined discrete predictions that agree with the dynamic proposed by the quantitative tool.

**Conclusions:**

MajS is a new method to test the consistency between a regulatory network and a dataset that provides computational predictions on unobserved network species. It provides fine-grained discrete predictions by outputting the weight of the predicted sign as a piece of additional information. MajS’ output, thanks to its weight, could easily be integrated *with* metabolic network modelling.

## Introduction

In recent years, there has been a sharp increase in our understanding of biological networks such as metabolic, signalling, and regulatory networks. For many organisms, the biological networks topology is already known, at least partially, and can be found in databases such as KEGG [1] or BIGG model repositories [2]. In parallel, gene expression data is also widely available in databases such as GEO [3] and provides us with gene expression profiles before and after a system perturbation. By system perturbation we refer to an over- or under-expressed node (or set of nodes) in the system compared to a control condition. The nodes can be either genes or proteins. Perturbations can be studied in the context of a disease, treatment, or environmental change.

For many years, metabolic and regulatory networks have been studied separately. The methods proposed to study them aim to simulate in silico the impact of some perturbation [4]. Regulatory networks, composed either by genes or proteins, can be modelled with Bayesian, neural or logic networks (Boolean or fuzzy logic), as well as with differential equations [5]. Metabolic networks are most of the time modelled using Flux Balance Analysis (FBA) [6]. Some regulatory network modelling tools use prior knowledge networks and gene expression data, extracted from independent sources, to understand the mechanisms triggered by a perturbation of a biological system [7]. Other tools such as [8] propose in silico experimental designs to discriminate regulatory network models. The idea is to use these approaches to compare network and data in order to propose in silico predictions which give novel insights on the biological system. Because of the incomplete, altered and noisy nature of biological data, it is expected that inconsistent behaviours appear upon network and data comparison. Some tools focus on the identification of such inconsistencies [9]. An inconsistent behaviour can be reflected by a missing interaction, an inaccurate observation or a wrongly defined logic of the interaction in the model. In some cases automatic repair of such inconsistencies is required to propose in silico predictions.

A gene and protein regulatory network can be related to the metabolic network via the enzymes produced by the regulatory network. Some of these enzymes have an impact on biochemical reactions within the metabolic network. In order to understand in detail the mechanisms behind a disturbance on the biological system, it appears essential to integrate these two types of networks.

In a previous study [10], we focused on the modelling of a regulatory network of the HIF-1 signaling pathway also called Hypoxia signaling pathway, which is of great interest in neurodegenerative diseases. We compared this regulatory network with Alzheimer’s disease gene expression data and we perturbed the system by inducing or repressing the HIF1A protein in silico. In order to allow us to predict the behaviour of unobserved species, the system was modelled using a logical and a Bayesian approach. We demonstrated that the logical approach, Iggy [7], was fast and reliable enough to predict unobserved nodes in the network upon system perturbation when compared to the Bayesian approach, Probregnet [4]. We have encountered, however, two issues that complicate the regulatory-metabolic network integration process. First, a quantification of Iggy’s qualitative predictions (in a three value domain) may introduce new biases to the entire modelling process. Second, because of the semantic of the *sign consistency* underlying Iggy’s modelling approach, the comparison uses relaxed rules that do not allow us to distinguish the computational predictions output from two types of in silico perturbations in this case-study.

We propose a novel logical approach using Answer Set Programming (ASP), named *MajS*, which addresses the previously mentioned difficulties. This approach, similar to Iggy, compares a regulatory network with gene-expression datasets, searches for inconsistencies, proposes minimal repairs and can predict unobserved nodes. It relies, however, on a different sign-consistency rule which takes into account the majoritarian sign of the nodes’ direct predecessors. As an output, added to the consistent sign of a node, it proposes weights which represent the confidence of the predicted sign. We are therefore able to more finely quantify the unobserved nodes. Also, because of the new semantic imposed, we are able to provide predictions more sensitive to the system perturbations. Furthermore, the predictions associated with their confidence weights provide new quantitative insights that make it possible to connect regulatory and metabolic models. Notice that this connection has not been explored in this study.

Our results show that MajS is more stable than Iggy concerning the coverage (the percentage of the number of predicted nodes against all unobserved nodes) of its predictions. In all our performed benchmarks Iggy’s coverage fluctuates between 20 and 100% while MajS is always 100%. Besides, MajS’ predictions are more sensitive to perturbation than Iggy’s. Indeed, for one of our benchmarks, Iggy outputs the same predicted sign upon different perturbations whereas MajS allows measuring the change of perturbation on predicted sign, thanks to the notion of weight. We also show that MajS has better accuracy of its predictions compared to in vitro perturbed data. Finally, MajS’ predictions’ dynamic trend agrees with the Bayesian approach predictions.

## Background

This section briefly describes the studied problem and then presents the Answer Set Programming (ASP) paradigm used by our method. It also introduces two alternative approaches, Iggy and Probregnet, which predict unobserved nodes in regulatory networks. These approaches are compared with MajS in the Section Results.

### Sign consistency modelling

The sign consistency approach tests the consistency between an interaction graph (IG) and a list of partial discrete observations of this graph nodes derived from experimental datasets. The IG is a signed directed graph, where the edges are signed as “+” or “−” and directed so that ${i} \rightarrow {j}$ means species *i* influences species *j*. The list of discrete observations is composed of discrete (“$+$”, over-expressed; “−”, under-expressed; 0, no-change) changes associated with some nodes. This change represents the differential expression of a gene between two system conditions (for example, normal and perturbed). Given a *sign consistency rule*, a graph is said to be consistent with respect to a list of discrete observations if the influences agree with the sign of observations for each node in the list. In case of inconsistency, the modelling framework proposes artificial repairs allowing to establish consistency. After consistency is established, the modelling agrees on new discrete changes on some initially unobserved species; these agreements are called predictions.

### ASP bases

ASP is a declarative programming language used to address combinatorial search problems. ASP can define logic programming rules, expressed using first-order logic, within a discrete domain, and find stable Herbrand models which satisfy these rules [11]. The logic program is written using a declarative programming paradigm: *what is to be solved, instead of how?*. This logic program is composed of the following ingredients:

- Generate: rules to generate the set of potential solutions.
- Test: rules to trim the set of potential solutions, eliminating unwanted ones.
- Define: (optional) rules to define auxiliary predicates.

For some problems, optimisation constructs are defined to find optimal solutions among a solution space. Thanks to ASP, it is also possible to handle intersection, union, enumeration and optimisation of models. In addition, unlike other declarative approaches (e.g. Prolog), ASP allows one to work with negation by default: a predicate is false as long as no indication allows to say that it is true.

### Iggy

Iggy [7]^1^ is a framework based on ASP that uses sign consistency modelling. Iggy automatically detects inconsistencies between graph and observations, applies minimal repairs to establish consistency, and predicts the sign of unobserved nodes by applying the following logical rules:

1. The observations must keep their initial sign.
2. The “+” or “−” sign for each signed node *n* must be justified by at least one of its received signed influences. An influence from node *p* to *n*, is the product between the (*p*, *n*) edge sign and *p*’s sign.
3. Each node signed as “0” must have only one influence signed as “0” or at least one “$+$” and one “−” influence.

Iggy proposes a set of consistent models. Then, Iggy summarises all consistent models in a step called Projection. Iggy has 6 different levels of predictions which are estimated after the Projection step: “−”, “notPlus”, “0”, “notMinus”, “+”, “CHANGE”. “−”, “0”, “+” are strong predictions as the node is always predicted with the same sign in all consistent models. “notPlus”, “notMinus”, “CHANGE” are soft predictions: a node can be predicted with different signs across all consistent models (“notPlus”: {“−”, “0”}; ‘notMinus”: {“+”, “0”}; “CHANGE”: {“+”; “−”} ). An example of Iggy’s application on a toy example is given in the Additional file 1.

### Probregnet

The Probregnet^2^ pipeline [4] is a framework that allows integration of a gene regulatory model (based on graph interactions) into a metabolic network (based on biochemical reactions) using an integer linear programming optimisation.

For this paper, we focus on the regulatory network analysis proposed by Probregnet. This analysis is based on Bayesian networks (BN) also called probabilistic directed acyclic models [12]. Probregnet’s input consists of both a regulatory network converted into a BN and gene expression data used to parametrise this BN. Probregnet needs at least ten samples (e.g. patients, cells or tissues) of gene expression data on one cellular condition or state to provide a complete parameterisation of all the graph nodes. Then, belief propagation is used on the BN to establish the repercussion of the perturbation of a given node in the graph over the other nodes.

## Results

We show in this section the results obtained after applying MajS (see section "Methods") on two case-studies. All scripts and data are available on GitHub: https://github.com/soph-lebars/MajS.

### Experimental setup

We focus on the regulatory network, modelled by an interaction graph (IG), and the impact of a perturbation on this regulatory network evaluated by discrete observations obtained from two gene expression datasets.

*Biological network - interaction graph* We focus on the regulatory network of the HIF-1 signalling pathway, known to be of importance in neurodegenerative diseases [13]. This graph was extracted from the KEGG database [1]. Nodes represent proteins or genes, and edges represent activations or inhibitions between two nodes. We reduce the regulatory network by keeping only nodes associated with expressed genes in the two datasets used in this study. The two networks, respectively reduced with Alzheimer’s disease (AD) and RNA-Seq datasets, are composed of 94 and 81 nodes and 285 and 233 edges.

*Datasets* We evaluate our model against two datasets composed of gene differential expression in two conditions. The Microarray dataset corresponds to cells from the hippocampus brain region [14]. It compares data from AD patients to data from Healthy individuals. The RNA-Seq dataset corresponds to HUVECS (Human umbilical vein endothelial cells) [15]. It compares the HUVECS response to an induced overexpression of HIF1A to one with a normal HIF1A expression.

*Benchmarks* We aim to study the impact of perturbing the system with a focus on the node HIF1A, a key protein of the HIF-1 signalling pathway. Recall that, one of the inputs of our method consists of a list of discrete observations for which a significant change of expression is detected between two conditions. The changes of expression our method accepts are: “+”, over-expression; “−”, under-expression; and “0”, no-change of expression. The values of the thresholds used to detect significant over- or under-expression are fixed according to the nature of each dataset as detailed below.

*Thresholds choice in HUVECS Benchmarks* For the RNA-Seq dataset, we used the logFC (log of gene expression) from cells with HIF1A in vitro over-expressed over normally expressed genes that were already provided in [15]. We use a threshold of 1.5 that is commonly used for logFC as said in [16]. The genes with logFC over 1.5 are set to “+”, the ones below $-1.5$ are set to “−”, and the ones between $-0.15$ and 0.15 are set to “0”. Using these thresholds, we obtain 30 observed nodes (out of 81 in the graph).

*Thresholds choice in AD Benchmarks* We aimed to study the impact of perturbation over HIF1A on the enzymes for the AD dataset. We used a threshold over the fold change distribution. The fold change is the expression of the gene in AD patients over the expression of the corresponding gene in Healthy individuals of all the genes in this dataset. The genes with FC that are over the third quartile are set to “+”; the ones under the first quartile are set to “−”; and the ones between 0.99 and 1.01 are set to “0”. Using these thresholds, we obtain 64 nodes (out of 96 in the graph) that compose the input observation list of our method. For the AD case, perturbations of HIF1A are only done in silico. We generate 3 different perturbations by adding the following observations to the list of 53 observations, described before: (plus) HIF1A=‘+’, (minus) HIF1A=‘−’, and (zero) HIF1A=‘0’.

### MajS applied to model HIF-1 signalling pathway and HUVECS dataset integration

#### Data

The IG for this case study is composed of 81 nodes and 233 edges derived from the HIF-1 signalling pathway and compared with a RNA-Seq dataset from HUVECS (see section "Experimental setup"). This IG is compared with two different lists of discrete observations; denoted by *Benchmark 1* and *Benchmark 2* in Table 1. Benchmark 1 is composed of 30 nodes that are a partial observation of the IG, generated by estimating significantly expressed genes in the RNA-Seq dataset using specific thresholds (see section "Experimental setup"). Benchmark 2, composed of 25 nodes, is a modification of Benchmark 1; where we have altered or removed the value of 9 observations, direct neighbours of HIF1A or directly linked to the network enzymes. These modifications were done to improve the coverage of Iggy. By modifying these observations, the problem becomes simpler to solve for Iggy, leading to a better coverage for Iggy. All these benchmarks are available on the GitHub companion repository.

#### MajS results on HUVECS dataset

On Benchmarks 1 and 2, MajS generates predictions for all initially unobserved nodes. MajS is configured by setting $K=3$ as the maximum artificial influences per node. The computations took approximately 97s.^3^ for each benchmark. MajS obtains 2016 optimal answer sets for both benchmarks, that is, 2016 assignments of nodes with a sign. The number of minimal artificial influences added by MajS to restore consistency in both benchmarks was 8. They are spread over seven repaired nodes and a maximum of 2 artificial influences per repaired node. The HIF-1 signalling IG is 2-consistent concerning the HUVECS dataset (see section "Consistency and repairs").

#### Comparison of MajS and Iggy

The aim of the sections from sections "Comparison of MajS and Iggy" to "Comparison of MajS and Iggy predictions with real data" is to understand the difference in prediction on the HUVECS dataset between MajS and Iggy. Iggy is described in section "Background" and in [10].

In Table 1, we show a global comparison of both tools with the two different benchmarks. For Benchmark 1, 51 nodes are unobserved in the IG. We can see that MajS was able to predict all of them ($100\%$ of coverage), whereas Iggy could predict only 30 nodes ($59\%$ of coverage). In order to compare the predictions’ signs for both methods, we consider for MajS the majoritarian sign of the predicted nodes. 22 nodes are predicted with the same sign for both methods, while 8 nodes are predicted differently between both methods. For Benchmark 2, we obtain for both methods $100\%$ of coverage. The number of predicted nodes in common is 48, and the number of predicted nodes different remains 8. Besides, these 8 nodes are the same for both benchmarks.

The different coverage obtained by MajS and Iggy in Benchmark 1, is explained by the different type of rule imposed to each graph node in both methods. Recall that Iggy implements a sign consistency rule stating that *a node sign has to be explained by at least one signed influence received*, whereas MajS implements a majoritarian sign rule stating that *a node sign has to be explained by the majoritarian sign of the influences received*. When a node receives a positive and a negative influence, Iggy cannot infer any prediction (both “+” and “−” scenarios are possible) whereas MajS will predict either 0, in case of balance, or the majoritarian sign. For that reason, MajS is always generating more predictions than Iggy.

This is illustrated by the coverage comparison in Benchmark 1.

**Table 1:** Table of comparison between Iggy and MajS for two benchmarks

	Benchmark 1		Benchmark 2	
	MajS	Iggy	MajS	Iggy
Predicted nodes	51	30	56	56
Coverage of predicted nodes	100.0 %	59.00 %	100.0 %	100.0 %
Number of predicted nodes: common VS different	22 VS 8		48 VS 8	

#### Different computational predictions for Iggy and MajS

In Table 2, we present the eight nodes predicted differently. The six nodes in bold are predictions for which there is an intersection between Iggy and MajS predictions. For example: (1) for PLCG1, the predicted sign of MajS, “−”, is included in the prediction “notPlus” of Iggy, and (2) for RBX1, the prediction of Iggy is included in the prediction of MajS. The two italic nodes in Table 2 refer to different predictions between Iggy and MajS. However, for these nodes, the number of cases that MajS predicted the same sign as Iggy remains high (672/2016) despite not being majoritarian.

To illustrate this prediction difference between MajS and Iggy, we can analyse PLCG nodes (PLCG1, PLCG2) in detail. MajS summarises all the optimal answer sets, so it outputs the majoritarian sign, its average weight and the standard deviation. For PLCG nodes, MajS gives “−” as the majoritarian sign, but we can see that “0” is also present in the optimal answer sets distribution. Iggy does not allow this distribution analysis and outputs “notPlus”, which signifies that there are “−” and “0” in the optimal answer sets but cannot allow determining the most representative one.

To conclude, MajS allows more information on predicted nodes than Iggy, and it outputs predictions that are, for most of the cases, with signs that often coincide with those predicted by Iggy. MajS outputs more detailed information than Iggy: number of answer sets and weight distribution.

**Table 2:** Table of different predicted nodes between Iggy and MajS

Name	SignMaj	Sign = “+”	Sign=“–”	Sign=“0 ”	SignIggy
**PLCG1**	−	[0, 0, 0]	[1344, 100, 0]	[672, 100, 0]	notPlus
**PLCG2**	−	[0, 0, 0]	[1344, 100, 0]	[672, 100, 0]	notPlus
**RBX1**	$0 / -$	[504, 100, 0]	[756, 100, 0]	[756, 100, 0]	0
**VHL**	$0 / -$	[504, 100, 0]	[756, 100, 0]	[756, 100, 0]	0
**RELA**	$+ / 0$	[756, 44, 16]	[504, 44, 16]	[756, 100, 0]	0
**NFKB1**	$+ / 0$	[756, 44, 16]	[504, 44, 16]	[756, 100, 0]	0
*IFNGR1*	$+$	[756, 100, 0]	[588, 100, 0]	[672, 100, 0]	0
*IFNGR2*	$+$	[756, 100, 0]	[588, 100, 0]	[672, 100, 0]	0

SignMaj refers to the majoritarian sign across all optimal answer sets. Columns 3–5 show the detailed distribution for each predicted sign across all optimal answers (in total 2016) sets according to MajS; the numbers in brackets refer to the number of answer sets where the node was fixed to this sign, the average weight and its standard deviation. SignIggy refers to the sign predicted by Iggy

#### Comparison of MajS and Iggy predictions with real data

Using a normal distribution mixture (see section "Comparison of discrete predictions with continuous values"), we compare the significance score of both methods to predict the real fold change data. This one is extracted from HUVECS dataset (see section "Experimental setup") where the perturbation was conducted in vitro. The aim of comparing this data with both methods is to see if they are able to model a perturbation in silico and have predicted fold change close to the fold change with an in vitro perturbation. This comparison is conducted as a validation.

We apply our method introduced in section "Comparison of discrete predictions with continuous values" using $\mu _{-}=-0.394$, $\mu _{0}=0$ and $\mu _{+}=0.489$ as parameters of the three normal distributions. These values are the respective means observed in the experimental HUVECS data using the thresholds fixed in section "Experimental setup". The standard deviation is calculated using $\sigma _{lc}=0.5$, which is approximately equal to the difference between two means (e.g., $\mu _{+}-\mu _{0}$ for instance), and $\sigma _{hc}=0.05$. As an illustration, the computed standard deviation is respectively 0.5 when $w=0$, 0.05 when $w=100$, and 0.275 when $w=50$. Notice finally, that all predictions provided by Iggy are to be considered with a high confidence weight, so they are assumed to have weight $w=100$ in our comparison.

**Fig. 1:**
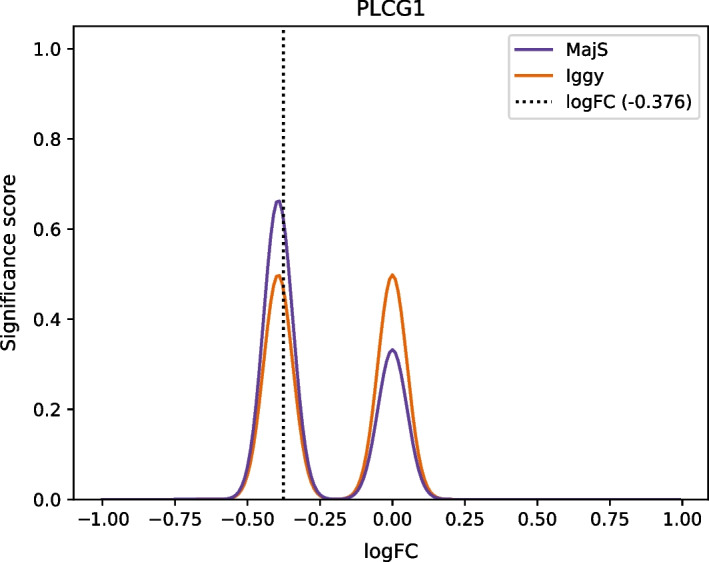
Iggy’s and MajS’ mixture of PLCG1 gene

For Benchmark 1, 21 unobserved genes (out of 51) are left out due to not being predicted by Iggy (see section "Comparison of MajS and Iggy"). If we focus, for example, on the PLCG1 gene prediction, MajS’s mixture provides a higher significance score than Iggy’s (Fig. 1). According to Eq. 2, the obtained mixture density function for MajS is

$$\begin{aligned} M_{MajS}(x)= \frac{1344}{2016} N_-(x) + \frac{672}{2016} N_0(x) + \frac{0}{2016} N_+(x), \end{aligned}$$

with $N_-(x)$, $N_0(x)$, and $N_+(x)$ being the probability density functions of three normal laws with different means and standard deviations (see section "Comparison of discrete predictions with continuous values" for details). Coefficients of these functions are the ratio of answer sets for which the node sign is predicted (see columns 3–5 of the Table  2, node PLCG1). The obtained mixture density function for Iggy is

$$\begin{aligned} M_{Iggy}(x)= \frac{1}{2} N_-(x) + \frac{1}{2} N_0(x) + \frac{0}{2} N_+(x), \end{aligned}$$

with the same density functions $N_-(x)$, $N_0(x)$ as for MajS in this example. Given the “notPlus” predicted sign for PLCG1 node (see SignIggy column in Table 2), corresponding to an equivalent prediction of “−” and “0”, the ratios for $N_-(x)$ and $N_0(x)$ density functions are equal to $\frac{1}{2}$.

According to Eq. 4 and to predict the log fold change value of PLCG1 ($-0.38$), the significance scores are equal to 0.62 for MajS and 0.47 for Iggy. Thus, MajS method provides a better prediction for the PLCG1 gene.

Considering the 30 initially unobserved genes of Benchmark 1, we found that MajS and Iggy produce the same mixture density for 14 genes, thus providing the same prediction. However, for the 16 other genes, MajS provides a better prediction for 10 out of 16 genes, in the sense that the computed score for MajS is greater than the one computed for Iggy. The same observations can be done for Benchmark 2; the computed prediction scores are equal for 16 genes; for 33 genes, MajS provides a higher score while Iggy provides a higher score for only 7 genes. This supports that MajS can obtain higher confidence on the predicted signs than Iggy does.

### MajS applied to model HIF-1 signalling pathway and Alzheimer’s disease (AD) dataset integration

This section focuses on the enzyme prediction to do the link with the metabolic network. Our first aim is to illustrate the difference in coverage and sensitivity between the two discrete approaches: MajS and Iggy, on a Microarray dataset of Alzheimer’s Disease patients. Our second aim is to compare MajS with a Bayesian quantitative approach, Probregnet, to point out the similarity of predictions in terms of dynamic evolution across different perturbations. Probregnet [4] predicts unobserved nodes with quantitative values in a regulatory network (see details in Section Background 5).

#### Data

The IG for this case study comprises 94 nodes and 283 edges. It was derived from the HIF-1 signalling pathway (see section "Experimental setup") and initially compared to three different lists of discrete observations. Each list is based on the *AD Benchmark* generated by estimating significantly expressed genes in the AD dataset using thresholds (see section "Experimental setup"). The three lists of observations were derived by fixing the value of node HIF1A to “+”, “−”, or “0” in order to simulate in silico a HIF1A perturbation. These three *original datasets* were composed of 64 observed nodes. When comparing the IG with these original datasets, Iggy provided new predictions for only 9 nodes (out of 30 unobserved ones), whereas MajS predicted the 30 nodes.

In order to provide a comparison of the predictions of MajS, Iggy and Probregnet for this case study, we modified the original datasets by fixing the value of HIF1A neighbours and removing observations referring to enzymes. In total, we performed 14 modifications. These modifications were done with a similar idea as for the HUVECS dataset (see Section 4.3.1), to improve Iggy’s prediction coverage. With the original dataset, Iggy predicted less than 30% unobserved nodes. Besides, observed enzymes are removed to see if the prediction agrees with the real dataset observations.

Our three resulting lists of observations were composed of 54 observations. We denote these three benchmarks as: *Benchmark_zero*, *Benchmark_plus*, and *Benchmark_minus* referring to HIF1A fixed to “0”, “+”, “−” respectively. The rest of this section is presented with these modified benchmarks.

#### MajS results on AD dataset

The computation took less than 40 s. for each benchmark. For *Benchmark_minus* we obtain 480 optimal answer sets with 32 repaired nodes, for *Benchmark_zero* we obtain 320 optimal answer sets with 27 repaired nodes and *Benchmark_plus* we obtain 160 optimal answer sets with 28 repaired nodes. The number of artificial influences K added for each repaired node by MajS to restore consistency was maximum 4. The IG is 4-consistent for the AD dataset (see section "Experimental setup").

#### Difference of coverage between MajS and Iggy across all benchmarks

MajS predictions’ coverage is 100% across all three benchmarks (40 unobserved nodes). For Iggy the coverage was of $20\%$, $88\%$, and $85\%$ for benchmarks having HIF1A set to “−”, “0”, and “+”, respectively. MajS has better coverage than Iggy for this case study.

#### MajS is more sensitive than Iggy to nodes perturbations

This section focuses on the 15 enzymes present in our IG; a more refined discrete prediction of these nodes may facilitate the IG model integration with a metabolic network model. Table 3 presents the computational predictions of MajS and Iggy on the enzyme nodes when comparing the IG with the three datasets of observations with different values (“−”, “0”, “+”) set for HIF1A. All MajS predictions were strong predictions (i.e., no variation across all optimal answer sets) with a unique weight.

For *Benchmark_minus*, Iggy could not predict the enzyme signs, while MajS was able to give a majoritarian sign of “0” associated with a weight of 100 to all the enzymes. This is explained by the different rule imposed to each node by Iggy (sign-consistency) and MajS (majoritarian sign). As found for the HUVECS dataset (see section "Comparison of MajS and Iggy"), Iggy constraints less the problem, generating more answer sets and producing fewer predictions.

For *Benchmark_zero* and *Benchmark_plus*, Iggy proposes similar predictions; consequently, it is not possible to observe any impact of the HIF1A perturbation. Instead, for MajS 12 enzymes are predicted as “+”, and they hold different weights (25 and 50 respectively) according to the benchmark. MajS is more sensitive than Iggy to perturbations on node HIF1A for this case study. It allows a measurable repercussion of the perturbation with different strengths for most enzymes. This is possible thanks to the weight term used in the domain of the answer sets obtained with MajS, and the weight assignment rule (see section "Test rules MajoritySign").

**Table 3:** MajS and Iggy predictions upon perturbations of HIF1A for 3 Benchmarks

	HIF1A$=$–		HIF1A$=$0		HIF1A$= +$	
Name	MajS	Iggy	MajS	Iggy	MajS	Iggy
**ALDOA**	(0,100)	Na	($+$,25)	$+$	($+$,50)	$+$
**ENO1**	(0,100)	Na	($+$,25)	$+$	($+$,50)	$+$
**ENO2**	(0,100)	Na	($+$,25)	$+$	($+$,50)	$+$
**ENO3**	(0,100)	Na	($+$,25)	$+$	($+$,50)	$+$
**GAPDH**	(0,100)	Na	($+$,25)	$+$	($+$,50)	$+$
**HK1**	(0,100)	Na	($+$,25)	$+$	($+$,50)	$+$
**HK2**	(0,100)	Na	($+$,25)	$+$	($+$,50)	$+$
**HK3**	(0,100)	Na	($+$,25)	$+$	($+$,50)	$+$
**LDHA**	(0,100)	Na	($+$,25)	$+$	($+$,50)	$+$
**PFKL**	(0,100)	Na	($+$,25)	$+$	($+$,50)	$+$
**PGK1**	(0,100)	Na	($+$,25)	$+$	($+$,50)	$+$
**SLC2A1**	(0,100)	Na	($+$,25)	$+$	($+$,50)	$+$
*PDHA1*	(0,100)	Na	$(-,100)$	−	$(-,100)$	−
*PDHA2*	(0,100)	Na	$(-,100)$	−	$(-,100)$	−
*PDHB*	(0,100)	Na	$(-,100)$	−	$(-,100)$	−

$Benchmark\_minus$ contained HIF1A=“–”; $Benchmark\_zero$ contained HIF1A=“0” and $Benchmark\_plus$ contained HIF1A=“+”. Here, “Na” means that Iggy could not predict for this Benchmark. MajS gives a predicted node as a tuple composed of the majoritarian sign and its average weight; the standard deviation is 0. The colours are focused on Benchmark_zero and Benchmark_plus; the enzymes predicted with “+” sign appear in bold. The ones predicted with “–” appear in italics

#### Comparison of MajS and Probregnet evolution of prediction of the 15 enzymes upon HIF1A perturbation

The focus here is on comparing the evolution of MajS’ predictions concerning the quantitative predictions of Probregnet [4] for the three types of HIF1A perturbations for the 15 enzymes. The repercussion of the HIF1A perturbation by Probregnet was monitored using the ratio, noted FC (fold change), between the node expression in the perturbed model (expression in AD patients) and the one in a non-perturbed model (expression in Healthy individuals).

**Fig. 2:**
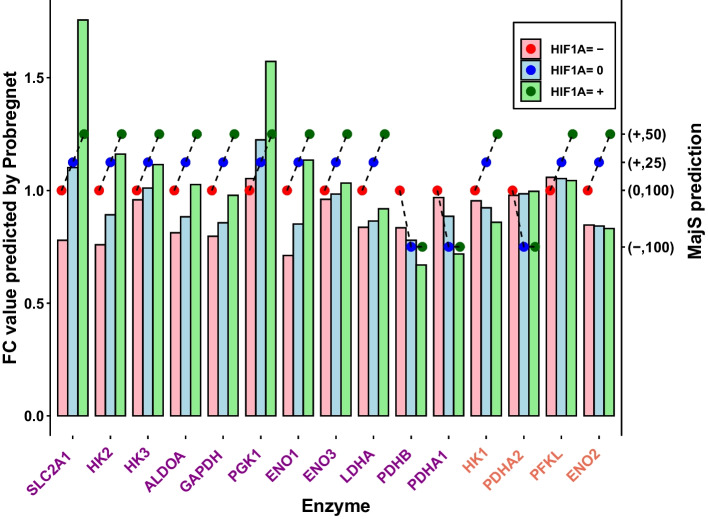
MajS and Probregnet enzymes predictions. For MajS, the evolution of prediction across the three HIF1A perturbations is shown with a dashed line and for Probregnet, with three consecutive bars for each enzyme. The left y-axis shows the foldchange (FC) predicted by Probregnet. On the right y-axis is the weighted label given by MajS. The x-axis shows the names of the enzymes. In purple, those that agree on evolution across perturbations between Probregnet and MajS. In orange, those with a disagreement on evolution

Figure 2 shows that 4 (out of 15) enzymes have a different evolution when comparing MajS to Probregnet. For MajS, the variation is measured by considering both the sign and the weight. According to MajS, all enzymes tend to increase in the transition from HIF1A=“−” to HIF1A=“+” except for PDH enzymes which tend to decrease. These variations agree with the IG topology; indeed, all enzymes are activated by HIF1A except PDH enzymes which are indirectly inhibited. According to Probregnet, 10 out of 15 enzymes are increasing (9/10 in agreement with MajS). In the 5 decreasing enzymes, there is a smaller proportion (2/5) of agreement with MajS. 3 out of 4 disagreements correspond to Probregnet enzyme predictions which are not significant, with a delta variation less than 0.02 when the average delta is 0.3 for the rest of the enzymes.

MajS and Probregnet give a similar dynamic trend for most enzyme predictions.

## Discussion and Conclusion

We present in our study a new logical approach, MajS, implemented in Answer Set Programming. MajS takes as input an interaction graph and a set of discrete observations. Discrete observations are expressed in the forms of “+”, “−”, or “0” signs in some of the graph nodes. This information is extracted from gene expression datasets. MajS tests the consistency between the majoritarian sign of a node’s direct predecessors and the node’s sign; detects and repairs inconsistencies, and predicts unobserved nodes. MajS’ prediction is given as a sign and a weight assigned to each unobserved node, where the weight represents the sign confidence. In addition, MajS outputs information concerning the prediction distribution across all consistent optimal answer sets or models. MajS was tested on two networks derived from the HIF-1 signalling pathway. These two networks were reduced with Alzheimer’s disease (AD) Microarray and HUVECS RNA-Seq datasets. They are composed of respectively, 94 and 81 nodes and 285 and 233 edges.

For both studied networks, MajS finds results in a couple of minutes which opens perspectives to handle larger networks. MajS outputs informative predictions on all unobserved nodes such as the majoritarian sign, the average weight, and the standard deviation of this weight in all benchmarks studied.

Several results are obtained upon comparison to a similar discrete and logical approach, Iggy. First, MajS’ coverage is higher than Iggy’s in all our tested benchmarks (see sections "Comparison of MajS and Iggy" and "Difference of coverage between MajS and Iggy across all benchmarks"). Second, MajS is more sensitive to the perturbation of our system thanks to the notion of weight (see section "MajS is more sensitive than Iggy to nodes perturbations"). Third, MajS is more reliable when compared to in vitro real data (see section "Comparison of MajS and Iggy predictions with real data"). With respect to Iggy’s implementation, MajS logical rules better constrain the network-data integration problem. Thus, MajS can propose a wider view, together with a distribution analysis, of all optimal answer sets. This is hard to accomplish with Iggy; for example, enumerating all optimal answer sets for the AD Benchmark (see section "Data") outputs $10^{10}$ solutions after 21 days without giving a complete solution.

We also compared MajS to a Bayesian approach, Probregnet. Focusing on the enzyme nodes prediction, MajS and Probregnet agree (11/15) on a similar dynamic regarding the evolution of enzyme predictions across HIF1A perturbation. The differences observed between MajS and Probregnet occur for enzymes for whom Probregnet prediction was not significantly varying across different perturbations (see section "Comparison of MajS and Probregnet evolution of prediction of the 15 enzymes upon HIF1A perturbation"). As with Iggy [10], MajS uses fewer input data than Probregnet. Also, Probregnet and MajS are used for specific purposes. Probregnet allows modelling small networks; whereas MajS and Iggy are adapted for larger networks. Both can measure the impact of a single node perturbation in a system. However, MajS can model multiple nodes’ perturbations. Probregnet works on a specific condition, whereas MajS deals with differential comparison between two conditions. Nevertheless, it is interesting to obtain quantitative predictions, as proposed by Probregnet, that easily adapt to linear programming metabolic modelling. MajS goes a step further than Iggy by outputting finer discretised predictions. A perspective of this work would focus on integrating MajS predicted enzymes into a metabolic network model and compare the results obtained with respect to Probregnet’s full pipeline. MajS, contrary to Probregnet, also handles better network inhibitions by relying on network topology. Indeed, Probregnet authors added a correction term in their publication to cope with inhibitions [4].

One of the possible limitations of MajS could be the repair process. Different repairs can be used in an interaction graph (e.g. remove, add or flip by changing the sign of edges; remove, add or flip nodes). In our study, we choose to add influences (positive or negative) to agree with the majority sign rules. Indeed, combining the repair process can appear to be a good idea, but it can become time-consuming in practice. Another limitation of MajS is that it keeps the sign of observed nodes even if they are inconsistent. In case of many inconsistent nodes, we should question the quality of the experimental data or the interaction graph. Because of these inconsistencies, the method may give predictions which are not relevant.

All in all, we have proposed MajS, a new method, fast and reliable, that tests consistency and predicts the change of expression on unobserved nodes (sign and weights) when comparing a regulatory network with a gene expression dataset. Our method is applied to perturbed data. In particular, we applied this method to test consistency between the HIF-1 signalling pathway and a HIF1A overexpressed dataset. Besides, Majs by predicting weights allows us to have a more refined prediction and proposes predictions which are sensitive to system perturbation.

## Methods

### MajS principle

MajS requires as input data an interaction graph (IG), whose edges are directed and labelled as *activation* or *inhibition*. It also requires a list of discrete observations on some IG nodes. This list is composed of discrete values (*colours* or signs) assigned to some of the IG’s nodes. These values measure the change-of-state of a graph’s node (gene or protein) between two specific conditions (e.g. 2 samples corresponding to 2 different biological conditions). The type of discrete assignments provided in the list of observations is: “+” (green) if the node is over-expressed, “−” (red) if under-expressed, and “0” (blue) if there is no change of expression between the two conditions. Not all the graph nodes are included in this list of observations.

**Fig. 3:**
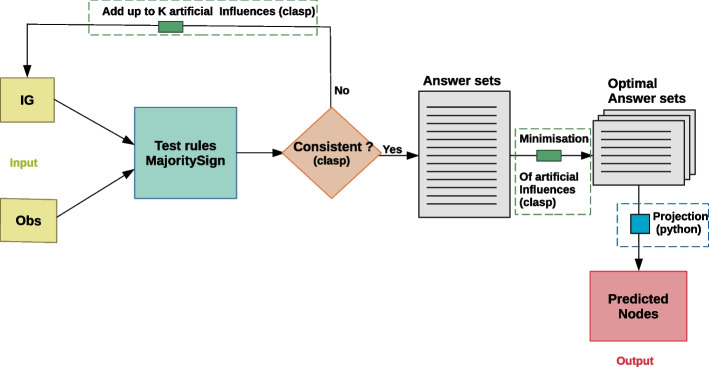
MajS workflow. In light green, we show the input data: the interaction graph, IG, and the discrete observation list, Obs. Then, we apply the logical rules implemented in MajS. We test the consistency and in case of inconsistency, we add artificial influences using K as a fixed parameter. That way we obtain answer sets that respect the logical rules. We minimise the artificial influences added to these answer sets and obtain an optimal subset of them. Finally, we project the optimal answer sets to obtain as output the predicted nodes of our model. clasp is the Answer Set Programming solver [17] used to implement most of MajS steps. Only the projection step was implemented in Python

We aim at predicting the sign (“−”, “0”, “+”) and weight (a score of confidence of the predicted sign between 0, for low confidence and 100, for high confidence) of unobserved nodes of the IG after it is compared to the list of observations. Prediction can be only computed in case of consistency between the IG topology and the gene expression data measurements. In order to establish consistency, we search for minimal repairs in the IG by adding artificial nodes to the graph. The number of minimal repairs is controlled by a third input *K* of our method. Our method workflow is detailed in Fig. 3. The following subsections aim at presenting in detail all the steps of the workflow.

#### MajS input data

**Interaction graph (IG)**An interaction graph is defined by a 3-tuple $(V, E,\sigma )$ where V is a set of nodes, E $\subset \{V \times V\}$ is the set of oriented edges and $\sigma : {\textrm{E}} \rightarrow { \{+,-\}}$ is a function of the edges where the plus sign represents an activation, and the minus sign represents an inhibition.

**Experimental observation (Obs)** A list of discrete observations where signs of some IG nodes are given by experimental measure. Generally, a pre-processing step of the experimental data by fixing thresholds for significant expression is required at this point. After the discretisation process, the observed nodes can take three different values: “+” for over-expressed nodes; “−” for under-expressed; and “0” for unchanged nodes.

These experimentally observed nodes belong to a set denoted *S*. In this study, we fix the weight of all experimental observation nodes to 100 which is the weight representing the maximal confidence.

#### Test rules MajoritySign

In the following section, we make explicit the logical rules that are applied on the IG and the discrete observation list to test consistency.

1. *Experimental observation signs are kept* We impose that the sign {“−”,“0”,“+”} of the experimental observations in *S* are kept.
2. *Signed majority wins* A node is signed “+” or “−”, following the majority sign from all its received influences in {“−”,“+”}.
3. *Balanced* A node is signed “0”, either if it only receives 0-influences or if it receives the same proportion of signed { “−”, “+”} influences.
4. *Weight assignment* Every node *v* of the graph is associated with a sign and a weight, which represents the score on its sign as follows:
  If $v \in S$ (experimental observations), its weight is fixed to 100.
  If *v* is inconsistent and has been repaired, then its weight is fixed to 0.
  If *v* is consistent, then the weight is the ratio between the sum of the parent’s weights, holding the majoritarian sign, and its number of parents.
  The sign-weight pair is denoted as a weighted label. Experimental observations can also be inconsistent after applying MajS rules.

We propose the two following definitions to clarify the next MajS steps.

##### Definition 1

(*weighted labelling*) A *weighted labelling* is defined as an operation which equips each node of an interaction graph $G=(V,E,\sigma )$ with a sign and a weight associated to this sign. Formally, a weighted label $(\mu ,\omega )$ on a set of nodes $U\subset V$ is defined as a function $U \rightarrow \{``-'',``0'',``+''\}\times [0,100]$, where $\mu (v)$ is a function assigning a *sign* to a node *v* in *U*, and $\omega (v)$ is a weight expressing the confidence of the sign.

More precisely, $\mu (v)$ can take three different values: “+” for over-expressed nodes compared to an initial condition; “−”, for under-expressed; and “0”, for unchanged nodes. Additionally, $\omega (v)$ varies between low confidence (0) and high confidence (100). A weighted labelling is said to be *complete* when it provides a weighted label to each node in *V* (*i.e.,* $U=V$). In MajS’ implementation, the signs are integer values set to $-1$ for “−”; 1 for “+” and 0 for “0” which allows the use of arithmetic operations on the sign values.

##### Definition 2

(influence) Given an interaction graph $G=(V,E,\sigma )$ and a node with a sign $\mu$ and a weight $\omega$, for each edge of G (*s*, *v*), we define an influence *I*(*s*, *v*) by:

$$\begin{aligned} I(s,v) = \sigma (s,v) \mu (s). \end{aligned}$$

An influence is a 0-*influence* if and only if $I(s,v)=0$. It is a positive influence if $I(s,v)=1$ and a negative one if $I(s,v)=-1$.

#### Consistency and repairs

**Fig. 4:**
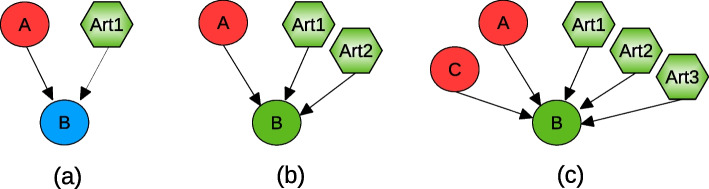
Inconsistencies between network topology and data. A species, gene, or protein, is represented as a node. The nodes’ colours represent the node’s sign when comparing two conditions. They are: blue, “0”, or no-change; red, “−”, or decrease; and green, “+”, or increase. The octagonal nodes represent the artificial influences added by MajS to restore conistency. For **a** an artificial influence, noted Art1 is added on node B with the sign “+” to balance with the sign of B’s successor node A. For **b**, two artificial influences, Art1 and Art2, are added to respect the majoritarian sign-consistency rule. Same logic for **c**

A graph is consistent if all of its nodes are consistent. A node is said to be consistent if its weighted label $(\mu ,\omega )$ is in adequacy either with its experimentally observed sign or with the logical rules application, i.e., the *signed-majority*, the *balanced*, and the *weight assignment* (rules 2, 3 and 4 in section "Test rules MajoritySign"). In case of inconsistency, MajS can repair the graph if its consistency can be established by adding artificial influences. A node is *K*-repairable when it was inconsistent and became consistent after adding at most *K* influences. A graph is *K*-consistent when all the nodes are at least *K*-repairable. Therefore, the problem is to determine, given an interaction graph *G*, an experimental observation set *S*, and an integer parameter *K*, if *G* is *K*-consistent. If this is the case, the minimal sets of repairs to recover consistency are identified. If not, the logical program is unsatisfiable. In Fig. 4 we show some examples of repair of the inconsistencies on node B.

#### From answer sets to optimal answer sets

After applying the logical rules and adding repairs in case of inconsistency, we obtain all the answer sets that respect the logical rules. These answer sets are a reduction of the *possible complete weighted labelling* presented in section "MajS search space". However, these solutions are not optimal as we did not minimise the number of possible repairs. Thus, an optimisation constraint is added to minimise the number of repairs. This constraint respects the logical rules in section "Test rules MajoritySign" so that it is guaranteed to find the minimal repairs to establish consistency.

#### Predicted nodes obtained after projection

After the optimisation step, many optimal (minimally repaired) answer sets can be proposed. All these solutions are consistent with the logical rules. In order to summarise these results, we add a step called projection. After this step, a node is assigned the following values computed after exploring all optimal solutions: a majoritarian sign (not necessarily unique), the average weight associated with the majoritarian sign, and the standard deviation of the weight. This triplet of values, assigned to all graph nodes, corresponds to the MajS predictions. Finally, MajS takes into account the added repairs in the weight assigned at this step: a node with a 0-weight associated with its sign implies it has been repaired.

The prediction can be either a strong or weak prediction; a strong prediction node means that its sign remains the same across all optimal answer sets and a weak prediction node means that its sign varies.

#### MajS application on a toy example

**Fig. 5:**
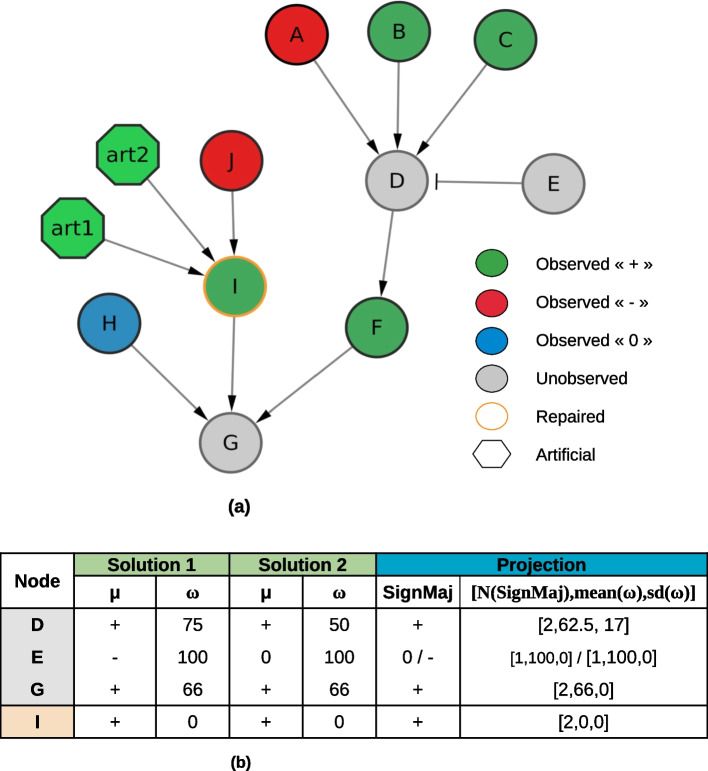
Toy case study. **a** Toy network with 7 nodes that are initially observed and 3 unobserved nodes. The *I* node is marked as inconsistent. **b** MajS predictions on toy network example. All unobserved nodes (grey) are predicted by MajS with a sign ($\mu$) and a weight ($\omega$). The orange node is repaired by adding two artificial influences. Columns Solution 1 and 2 represent sign and weight in optimal answer sets for unobserved and repaired nodes. Column Projection is summarizing all Solution columns as explained in section "Predicted nodes obtained after projection"

This section presents the results obtained while applying MajS on a toy example, an IG composed of 10 nodes, 7 activation edges, and 1 inhibition edge ($E \dashv D$). In Fig. 5 we illustrate how MajS proceeds when comparing this toy IG with one dataset of observations. First, MajS adds two artificial influences (art1 and art2) on node *I* to recover consistency. Then, it predicts values over nodes *D*, *E*, and *G*. In Fig. 5, we show the prediction of MajS for nodes *D*, *E*, *G*, and the repaired node, *I*. This toy example outputs two optimal answer sets: *Solutions 1* and *2*.

Focusing on node *D*, we observe that in the answer set *Solution 1*, the predicted weight is 75, while in *Solution 2*, the predicted weight is 50. Node *D* has 4 parents: 3 observed ($A=$“−”, $B=$“+”, $C=$“+”), and an unobserved parent *E*. To comply with the *Signed majority wins* rule (see section "Test rules MajoritySign"), *E* can be assigned either to “−” or “0”. Both assignments give a majoritarian “+” sign on *D*. When *E* is set to “−”, the weight of the “+” sign on *D* is 75 as it is defined as the percentage ratio between the sum of the positive influences and the total number of parents of *D*. A similar reasoning when *E* is set to “0” leads to the weight of 50 assigned to the “+” sign for *D*. Notice that no answer set proposes an assignment of *E* to “+”. If that was the case, *D*’s sign would be “0” (*Balanced* rule in section "Test rules MajoritySign") and would not explain the sign of its direct successor *F*; this assignment requires adding another repair and would not be an optimal solution anymore.

To illustrate how projections work (see section "Predicted nodes obtained after projection"), let us focus on nodes *D* and *E*. For node *D*, the sign across all optimal solutions is “+” so the majoritarian sign given by the projection computation is of “+” (Fig. 5b, column *Projection*, left sub-column *SignMaj*). We also show a detailed view of how this majoritarian sign is represented across all optimal answer sets. First, the number of optimal answer sets having the majoritarian sign for node *D* is 2. Second, the average weight associated with this sign for *D* is 62.5. Third, the standard deviation of the average weight is 17. These three values appear represented as a triplet (Fig. 5b, column *Projection*, right sub-column). Following the same logic, for node *E*, we have two different majoritarian signs: “0” and “−”, equally distributed across all optimal answer sets, both average weights are 100, and there is no standard deviation.

Finally, in Fig. 5b, we see that for node *I*, the weight is fixed to 0 by MajS in all optimal answer sets, implying that it was repaired. Indeed, *J* is the only predecessor of *I* and activates it. Thus, *J* and *I* would have the same sign in a consistent local behaviour. To guarantee a global consistency of the whole network, *I* should be repaired.

The inputs of the logical program for the toy case study can be found on GitHub: https://github.com/soph-lebars/MajS/tree/main/toycasestudy.

### MajS search space

After having the problem defined, we generate the *choice rules* constructs of our logic program. These rules define the solution space of a problem and create the candidates that are later filtered with the constraints.

*Possible complete weighted labellings* A complete weighted labelling is composed by both a sign function $\mu : V\rightarrow \{``-'',``0'',``+''\}$ and a weight function $\omega : V\rightarrow [0,100]$. We also assume that the interval [0, 100] is discretised by the set of integers $\{0,\dots ,100\}$, in that case, the weight is simply rounded to the closest integer. Thus, the total number of possible complete weighted labelling is equal to $3^{|V|}\times 101^{|V|}$.

*Possible repairs* Recall that *K* is a parameter given as input to the method. For each inconsistent node $v \in V$ we generate multiple sets of *k* artificial nodes and influences, with $k \le K$. Let us name this set as $p^{(v,k)} = \{p_1, \ldots , p_k\}$. The search space *P* is defined by the union of all possible ways to assign parents for each node in *V*, that is:

1 $$\begin{aligned} P = \bigcup _{v \in V, 1 \le k \le K} p^{(v,k)} \end{aligned}$$

An artificial parent $p_i$ interacts with *v* with a positive or negative influence, i.e., $I(p_i,v) \in \{-1,1\}$. Each $p_i$ is added to the graph *G* and its influence changes the computation of the majoritarian sign for node *v* (see rule 2 in section "Test rules MajoritySign").

### Different and common points between MajS and Iggy

We summarise the main differences and similarities between MajS and Iggy in Table 4.

The common points are that the two tools use the ASP paradigm to describe the inputs of the logical program, also called an instance, and to encode the logical constraints of the problem. Secondly, the optimisation of the problem is the same. Both methods seek to minimise the number of influences added to repair.

The two methods are different in three ways. First, the solution space for MajS will be larger because the sign of the nodes is also associated with a weight ranging from 0 to 100. The second difference is that Iggy adds only one influence per node to fix inconsistencies, whereas MajS adds several influences to reestablish consistency. The third difference is in the logical rules, which are implemented differently. Indeed, Iggy constrains the solution space using sign consistency: the sign of a node is consistent if it can be explained by at least one influence. Whereas, for MajS, the sign of a node is explained by the majority of influences received. In addition, MajS also uses logic rules to constrain the weight. The last difference is in the projection step, which allows obtaining the nodes predicted by MajS and Iggy by summarising the complete list of optimal answer-sets into predictions. For Iggy, the nodes are predicted according to the six values shown in Table 4. MajS summarises the optimal answer sets by predicting the nodes with their majoritarian sign, their associated weight and the associated standard deviation.

**Table 4:** Different and common points between Iggy and MajS

	Iggy	MajS
Instance (input)	$\bullet$ An interaction graph, whose edges are directed and labelled as *activation *or *inhibition*. $\bullet$ A list of discrete observa- tions on some IG nodes.	$\bullet$ An interaction graph, whose edges are directed and labelled as *activation *or *inhibition*. $\bullet$ A list of discrete observa- tions on some IG nodes.
Search space/ Guess	$$\bullet$$ Depends on node, sign $$\in$$ {“ − ”, “0”, “ $$+$$ ”} (3$$^{|V|})$$ $$\bullet$$ 1-influence repair added by node	$$\bullet$$ Depends on node, sign $$\in$$ {“ − ”, “0”, “ $$+$$ ”} , weight $$\in$$ [0, 100] (3$$^{|V|} \times 101^{|V|})$$ $$\bullet$$ K-influences repair added by node
Logical rules	$\bullet$ Experimental observation signs are kept. $\bullet$ A node signed as “0” must receive only one influence signed as “0” or at least one “$+$” and one “−” in- fluence. $\bullet$ A signed node must be jus- tified by at least one signed influence.	$\bullet$ Experimental observation signs are kept. $\bullet$ A node is signed “0” ei- ther if it only receives 0- influences or the same pro- portion of signed “ −”, “ $+$ ” influences. $\bullet$ A node is signed following the majority sign from all its received influences.
Optimisation	$\bullet$ Minimise the number of added repairs	$\bullet$ Minimise the number of added repairs
Projection (predicted nodes)	$$\bullet$$ Six levels of possible pre- diction: 1 - 2 notPlus (-, 0) 3 0 4 notMinus (0, $$+)$$ 5 $$+$$ 6 CHANGE ($$+$$, -)	$$\bullet$$ Majoritarian sign $$\bullet$$ Statistical information on the weight distribution (average, standard devia- tion)

### Comparison of discrete predictions with continuous values

Here, we describe our method for comparing the discrete predictions provided by MajS and Iggy to the continuous experimental values of fold change. We rely on mixtures of normal distribution which is proven to be a probabilistic model of choice for microarray experiments [18]. For each predicted node, we define a continuous probability distribution $\mathcal {M}$, whose density function is denoted as *M*(*x*) with $x\in \mathbb {R}$, that is a mixture of three Normal distributions whose means depend on the sign of the prediction and whose standard deviations depend on the weight of the prediction. Precisely, for $x\in \mathbb {R}$ the mixture density function is defined by

2 $$\begin{aligned} M(x) = \sum _{s\in \{-,0,+\}} \phi _s\cdot N_s(x), \end{aligned}$$

where $\phi _s$ is the ratio of answer sets for which the node sign is predicted as $s$ for $s\in \{-,0,+\}$, and $N_s(x)$ is the density function of a Normal distribution $\mathcal {N}(\mu _s,\sigma _s)$ with mean $\mu _s$ and standard deviation $\sigma _s$. Precisely, one has

$$\begin{aligned} N_s(x) ={\frac{1}{\sigma _s{\sqrt{2\pi }}}}{\text {e}} ^{-{\frac{1}{2}}\left( {\frac{x-\mu _s}{\sigma _s}}\right) ^{2}}. \end{aligned}$$

In our comparison, we fix mean values using experimental HUVECS data (see section "Comparison of MajS and Iggy predictions with real data" for more details). The standard deviation is derived from the weight $w\in [0,100]$ of the sign $s$ for a given node by using a simple linear transformation rule $\sigma _{hc}\cdot \frac{w}{100}+\sigma _{lc}\cdot (1-\frac{w}{100})$. Here, $\sigma _{lc}$ is a fixed constant considered as a low confidence prediction (which is assigned when $w=0$) and $\sigma _{lc}$ is a fixed constant considered as a high confidence prediction (which is assigned when $w=100$).

To compare MajS and Iggy’s methods, once we have a (mixture) density function *M*(*x*), we calculate:

3 $$\begin{aligned} {P}(fc) = {Prob}\{|F-fc|<\varepsilon \} = \int _{fc-\varepsilon }^{fc+\varepsilon } M(x) dx, \end{aligned}$$

where $\varepsilon$ is fixed to an arbitrary value of 0.005. Different values of $\varepsilon$ have been tested leading to similar results (see Additional file 1).

In order to improve the significance of *P*(*fc*), we compute the maximum value that can be reached by any mixture obtained within these settings. It is straightforward that the mixture that provides the maximum value is the one corresponding to a single prediction, say “0”, with weight 100. The maximum for *P*(*x*) is then reached for $x=0$. Consequently, the maximum value, denoted as $\mathcal {P}_{max}$ equals:

$$\begin{aligned} \mathcal {P}_{max} = \int _{-\varepsilon }^{+\varepsilon } {\frac{1}{\sigma _{hc} {\sqrt{2\pi }}}}{\text {e}} ^{-{\frac{1}{2}}\left( {\frac{x}{\sigma _{hc} }}\right) ^{2}} dx = 0.07969 \end{aligned}$$

For more details, we refer the reader to Additional file 1. Finally, we define a significance score ${\mathcal {S}}(fc)$ between 0 and 1:

4 $$\begin{aligned} {\mathcal {S}}(fc) = {P}(fc)/\mathcal {P}_{max} \end{aligned}$$

We use this significance score to compare both Iggy and MajS methods.

## Supplementary Information

**Additional file 1. Section 1**. Iggy application on toy example, **Section 2.** Computation of $\mathcal {P}_{max}$, **Section 3**. Impact of parameters $\varepsilon$, $\sigma _{hc}$, and $\sigma _{lc}$.

## Data Availability

All data and scripts are available on GitHub: https://github.com/soph-lebars/MajS.

## Abbreviations

FC: Fold change
IG: Interaction graph
AD: Alzheimer’s disease
ASP: Answer set programming

## Footnotes

1 http://bioasp.github.io/iggy/.

2 https://github.com/hyu-ub/prob_reg_net.

3 solver: clingo version 5.5.0, parallel execution on 10 cores. All computations are performed on a standard laptop machine. Ubuntu 18.04, 64 bits, intel core i7-9850 H CPU 2.60 GHz, 32 GB.
