# Supplementary material for: Predicting weighted unobserved nodes in a regulatory network using answer set programming
Source: BMC Bioinformatics. 2023 Aug 25;24(Suppl 1):321. doi: 10.1186/s12859-023-05429-3 (PMC10463596; doi:10.1186/s12859-023-05429-3)
Supplement: Supplementary file 1 — Additional file 1. Section 1. Iggy application on toy example, Section 2. Computation of $\mathcal {P}_{max}$Pmax, Section 3. Impact of parameters $\varepsilon$ε, $\sigma _{hc}$σhc, and $\sigma _{lc}$σlc. [file 12859_2023_5429_MOESM1_ESM.pdf]

Predicting weighted unobserved nodes in a regulatory network using Answer Set Programming

Sophie Le Bars[1], Mathieu Bolteau[1], Jérémie Bourdon[1] and Carito Guziolowksi[1]

[1] Nantes Université, École Centrale Nantes, CNRS, LS2N, UMR 6004, F-44000 Nantes, France.

1 Iggy application on a toy example

The toy example is the same as the one presented in Section 3.1.6 of the main text for the MajS application and is composed of 10 nodes, 7 activation edges, and 1 inhibition edge ($E \dashv D$). In Figure 1 we illustrate how Iggy proceeds when comparing this toy IG with one dataset of observations. First, Iggy recovers consistency by adding only one artificial influence (art) on node I. Then, it predicts values over nodes D, E, and G, which are unobserved. Figure 1 shows the prediction of Iggy for the three unobserved nodes and the repaired node, I. This toy example outputs three optimal answer sets : *Solutions 1*, *2* and *3*. Focusing on node E, we observe that in the answer set *Solution 1*, the predicted sign is "-", in *Solution 2*, the predicted sign is "+" and in *Solution 3*, the predicted sign is "0". Node D and G are always set to "+" as explained by the sign of their received influence (see rule 2 in Section 2.3 of the main text). To illustrate how projection works with Iggy, let us focus on nodes D and E. For node D, the sign across all optimal solutions is "+" so the sign given by the projection is of "+" (Figure 1 (b), column *Prediction*). For node E, the sign varies between $-$, $+$ and 0 across all optimal solutions ; in this case, Iggy cannot give a prediction. Finally, in Figure 1 (b), we see that the sign of node I is the same as its observed sign. Indeed, the added artificial influences allow the node to keep the observed sign despite the inconsistency. The inconsistency is explained by J, which is the only predecessor of I and activates it. Thus, J and I would have the same sign in consistent local behaviour. To guarantee a global consistency of the whole network, I had to be repaired.

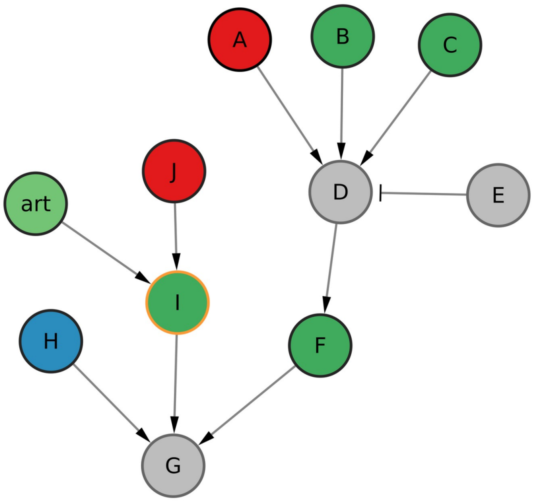

(a)

Node	Solution 1	Solution 2	Solution 3	Prediction
D	+	+	+	+
E	-	+	0	NA
G	+	+	+	+
I	+	+	+	+

(b)

FIGURE 1 – **Toy case study Iggy**. (a) Toy network with 7 nodes that are initially observed and 3 unobserved nodes. The I node is marked as inconsistent. (b) Iggy predictions on toy network example. Unobserved nodes (grey) are predicted by Iggy with a sign. The orange node is repaired by adding one artificial influence. Columns Solution 1, 2 and 3 represent sign in optimal answer sets for unobserved and repaired nodes. Column Prediction is summarizing all Solution columns.

2 Computation of $\mathcal{P}_{max}$

In Section 3.4 (Comparison of discrete predictions with continuous values) of our paper, we introduce a method allowing to compare discrete values obtained by Iggy or MajS methods to continuous values that come from experimental data. We compute a mixture density function $M(x)$ (Equation 2) and calculate the probability to predict a fold change using this mixture (Equation 3). To

determine if $P(fc)$ is relevant, we use the maximum value possible that can be taken by P for any mixture and any fold change value. The obtained value is denoted $\mathcal{P}_{max}$. It corresponds to the best fold change fc (equals to 0) for a mixture which predicts only this fold change with a weight of 100. The mixture exactly corresponds to the following MajS prediction :

— sign = "+", ♯Answer sets = 0, weight = 0
— sign = "0", ♯Answer sets = 1, weight = 100
— sign = "−", ♯Answer sets = 0, weight = 0

In this case, the mixture reduces to a normal distribution with mean 0 and standard deviation σ_{hc} :

$$M(x) = \frac{1}{\sigma_{hc}\sqrt{2\pi}}\, \mathrm{e}^{-\frac{1}{2}\left(\frac{x}{\sigma_{hc}}\right)^2}$$

This allows us to derive the maximum value $\mathcal{P}_{max}$:

$$\mathcal{P}_{max} = \int_{-\varepsilon}^{+\varepsilon} \frac{1}{\sigma_{hc}\sqrt{2\pi}}\, \mathrm{e}^{-\frac{1}{2}\left(\frac{x}{\sigma_{hc}}\right)^2} dx = 0.07969,$$

when $\varepsilon = 0.005$ and $\sigma_{hc} = 0.05$. This value depends on both σ_{hc} and ε. The impact of these constant parameters is discussed below.

3 Impact of parameters ε, σ_{hc} and σ_{lc}

Our method for computing a significant score relies on a few number of parameters. The first one is the ε parameter, used to compute the area under the curve of the distribution. We also use two parameters (σ_{hc} and σ_{lc}) in order to transform the weight given by MajS into a standard deviation involved in the normal distribution calculation. To observe the impact of these arbitrary choices on the significance scores and the comparison of MajS and Iggy methods, we make some tests with different values of these parameters. All the experiments are available on the GitHub repository.

3.1 Epsilon parameter

We lead some experiments to observe the impact of this arbitrary choice on the conclusion we make regarding the comparison of scores of both methods MajS and Iggy. We define a threshold $\mathcal{E}$ with $\varepsilon \leq \mathcal{E}$ where, for all values of ε, no change is observed with respect to the conclusion of the comparison of both methods (*i.e.,* a score of a method become better than the other). We test different values of $\varepsilon = \{0.001, 0.002, ..., 0.1\}$ in order to find a $\mathcal{E}$ adapted to our data. This experiment concludes that only two genes have different behaviour. Finally, we can deduce a value of $\mathcal{E} = 0.015$. In our study, we take $\varepsilon = 0.005$ that is far below $\mathcal{E}$. Notice that it is not the choice of epsilon that matters, but the conclusion of the comparison of scores of the two methods.

3.2 Low and high confidence parameters

Two parameters are fixed for the weight transformation into the standard deviation of the normal distribution. The objective of this transformation is to attribute a small standard deviation value when the weight is high, referring to a high confidence in the prediction. In contrast, the weight is transformed to a high value when it represents a low confidence in the prediction. In this case, the distribution mixtures are flattened. We test different values of high confidence ($\sigma_{hc} = \{0.01, 0.02, ..., 0.1\}$) and low confidence ($\sigma_{lc} = \{0.1, 0.2, ..., 1\}$) parameters. Like the preceding experimentation, we look at the difference in the scores of the both methods. We can identify some combinations of parameters values where no change is observed (FIGURE 2). Indeed, we deduce an interval I for σ_{hc} and σ_{lc} where the number of genes remains constant, thus leading to the same conclusions when comparing Iggy and MajS : $I_{\sigma_{hc}} = [0.05, 0.1]$ and $I_{\sigma_{hc}} = [0.5, 0.8]$. In our study, we fix $\sigma_{hc} = 0.05$ and $\sigma_{lc} = 0.5$.

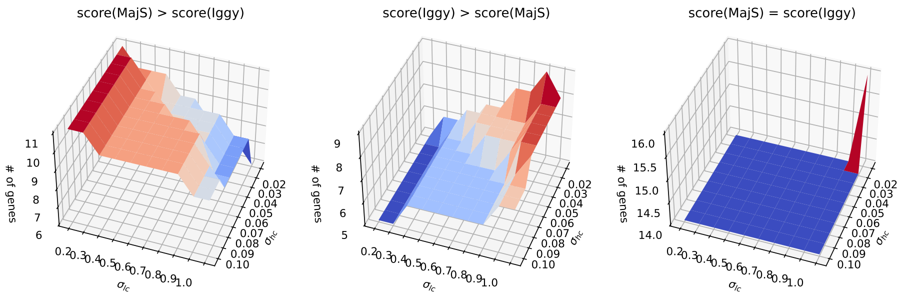

FIGURE 2 – Representation of the number of genes relevant in a specific case (score(MajS)>score(Iggy), score(Iggy)>score(MajS), score(MajS)=score(Iggy)) according to different values of σ_{hc} and σ_{lc}. This experimentation concerns the Benchmark1.
